# Inhibition of TLR4 Signaling Affects Mitochondrial Fitness and Overcomes Bortezomib Resistance in Myeloma Plasma Cells

**DOI:** 10.3390/cancers12081999

**Published:** 2020-07-22

**Authors:** Cesarina Giallongo, Daniele Tibullo, Fabrizio Puglisi, Alessandro Barbato, Nunzio Vicario, Daniela Cambria, Nunziatina Laura Parrinello, Alessandra Romano, Concetta Conticello, Stefano Forte, Rosalba Parenti, Angela Maria Amorini, Giuseppe Lazzarino, Giovanni Li Volti, Giuseppe Alberto Palumbo, Francesco Di Raimondo

**Affiliations:** 1 Department of Medical and Surgical Sciences and Advanced Technologies “G.F. Ingrassia”, University of Catania, 95123 Catania, Italy; giuseppealberto.palumbo@gmail.com; 2 Section of Biochemistry, Department of Biomedical and Biotechnological Sciences, University of Catania, 95123 Catania, Italy; d.tibullo@unict.it (D.T.); amorini@unict.it (A.M.A.); lazzarig@unict.it (G.L.); 3 Division of Hematology, Azienda Ospedaliero Universitaria, Policlinico Vittorio Emanuele, 95123 Catania, Italy; puglisi.fabri@gmail.com (F.P.); alessandrobarbato93@libero.it (A.B.); cambriad@tiscali.it (D.C.); laura.parrinello@tiscali.it (N.L.P.); sandrina.romano@gmail.com (A.R.); ettaconticello@gmail.com (C.C.); diraimon@unict.it (F.D.R.); 4 Section of Physiology, Department of Biomedical and Biotechnological Sciences, University of Catania, 95123 Catania, Italy; nunziovicario@unict.it (N.V.); parenti@unict.it (R.P.); 5 Fondazione “Istituto Oncologico del Mediterraneo”, 95029 Catania, Italy; stefano.forte@grupposamed.com; 6 Division of Hematology, Department of General Surgery and Medical-Surgical Specialties, University of Catania, 95123 Catania, Italy

**Keywords:** TLR4, myeloma, bortezomib resistance, mitochondria, refractory CD138+

## Abstract

Multiple myeloma (MM) is a B-cell malignancy requiring inflammatory microenvironment signals for cell survival and proliferation. Despite improvements in pharmacological tools, MM remains incurable mainly because of drug resistance. The present study aimed to investigate the implication of Toll-like receptor 4 (TLR4) as the potential mechanism of bortezomib (BTZ) resistance. We found that TLR4 activation induced mitochondrial biogenesis and increased mitochondrial mass in human MM cell lines. Moreover, TLR4 signaling was activated after BTZ exposure and was increased in BTZ-resistant U266 (U266-R) cells. A combination of BTZ with TAK-242, a selective TLR4 inhibitor, overcame drug resistance through the generation of higher and extended oxidative stress, strong mitochondrial depolarization and severe impairment of mitochondrial fitness which in turn caused cell energy crisis and activated mitophagy and apoptosis. We further confirmed the efficacy of a TAK-242/BTZ combination in plasma cells from refractory myeloma patients. Consistently, inhibition of TLR4 increased BTZ-induced mitochondrial depolarization, restoring pharmacological response. Taken together, these findings indicate that TLR4 signaling acts as a stress-responsive mechanism protecting mitochondria during BTZ exposure, sustaining mitochondrial metabolism and promoting drug resistance. Inhibition of TLR4 could be therefore be a possible target in patients with refractory MM to overcome BTZ resistance.

## 1. Introduction

Multiple myeloma (MM) is a hematological malignancy characterized by the accumulation of tumor plasma cells in the bone marrow (BM). Survival of MM patients has improved with the introduction of the proteasome inhibitors (PI) and immunomodulatory drugs. However, MM remains incurable and relapses and disease progression are common among MM patients [1]. Several mechanisms are suggested to be responsible for disease progression and Toll-like receptors (TLRs) seem to play an important role with particular regard to the microenvironment molecular mechanisms, TLRs are potential linking elements between inflammation and cancer [2]. They are a family of transmembrane receptors playing a pivotal role in sensing and initiating innate immune response recognizing exogenous pathogen-associated molecular patterns (PAMPs). TLR also have endogenous ligands such as heat shock proteins, fragments of extracellular matrix proteins, modified lipids, or lipoproteins (called damage-associated molecular patterns, DAMPs) [3,4]. Their signaling cascades via interactions with the TIR (Toll-interleukin-1 receptor) domains is mediated by specific adaptor molecules, including MyD88, TIRAP, TRIF and TRAM [5], leading to activation of NFkB and ERK/JNK/p38, which regulate many immunologically relevant proteins and are also involved in tumorigenesis and tumor growth [6]. Evidence from recent studies suggests that enhanced expression of TLR4 in chronic infectious and inflammation are correlated with cancer progression [4,7]. Indeed, aberrant activation of the TLR4 pathway induces an inflammatory pathway that promotes the initiation and progression of several tumors [8,9,10,11]. Gene expression microarray analysis of a large panel of MM patients revealed that TLR4 is overexpressed in approximately 6% of patients and is associated with a poor outcome [12]. TLR4 promotes MM cell proliferation partially through autocrine IL6 signaling [13,14] and induces PDL-1 expression through MyD88/TRAF6 pathway contributing to T-cell dysfunction and immune escape [15,16]. Moreover, we recently demonstrated that TLR4 signalling plays a key role in mesenchymal stromal cells (MSC) transformation by inducing a proinflammatory phenotype associated with a protumor behaviour, allowing immune escape mechanisms and tumor growth in the myeloma microenvironment [17].

BTZ, a proteasome inhibitor (PI) commonly used for the treatment of myeloma, selectively induces apoptosis in MM PCs by triggering endoplasmic reticulum (ER) stress [18,19,20]. Indeed, one of the features of plasma cells is an expansive and highly developed ER, essential for their secretory function [21]. BTZ rapidly induced components of the proapoptotic/terminal unfolded protein response (UPR) by inhibiting the retrograde translocation of misfolded proteins from the ER [21]. ER stress-induced apoptosis engages mitochondrial depolarization, cytochrome c release and its downstream caspase-9 activation. It has recently been demonstrated that the TLR4 pathway provides a protective effect against BTZ-induced ER stress and the pretreatment of MM cells with lipopolysaccharide (LPS), a TLR4 agonist, significantly reduces BTZ efficacy [22]. Several studies suggest that mitochondrial fitness may, at least in part, drive chemoresistance in cancer [23]. Accordingly, induction of an energy metabolism shift toward a low oxidative phosphorylation system (OXPHOS), by targeting mitochondrial functions, markedly enhanced the antileukemic effects of chemotherapy in acute myeloid leukemia [24]. Bioenergetic changes have also been implicated in the PI resistance of MM cells [25,26]. These adaptations are characterized by an increase in mitochondrial biomass and an increased reliance on mitochondrial respiration [26]. Increased OXPHOS has been found in MM cells from both relapsed and drug resistant patients [27]. Recently, we characterized the biochemical, metabolic and molecular features of a U266 clone resistant to BTZ (U266-R), both under resting conditions and after BTZ exposure [23,28]. In comparison to the sensitive clone (U266-S), U266-R based their resistance to BTZ on changes of various genes and protein expressions, several of which are involved in mitochondrial dynamics and protein glycosylation. Metabolically, U266-R are characterized by high levels of triphosphate nucleosides (ATP, UTP, GTP, CTP), ATP/ADP ratio and compounds of the hexoseamine biosynthetic pathway (HBP) indicating, respectively, sustained energy metabolism, efficient mitochondrial phosphorylating capacity and a high rate of protein glycosylation.

Considering the relationship between PI, ER stress and mitochondria, it is important to understand the stress-responsive mechanisms protecting mitochondria during ER stress contributing to drug resistance. As TLR4 activation in myeloma cells suppresses ER stress, reducing the efficacy of BTZ [22], we evaluated herein the role of TLR4 signalling on MM cells’ mitochondrial dynamics and energy metabolism as the potentially active mechanism of drug resistance to BTZ.

## 2. Results

### 2.1. TLR4 Activation Induced Mitochondrial Biogenesis in MM Cell Lines

**Figure 1:**
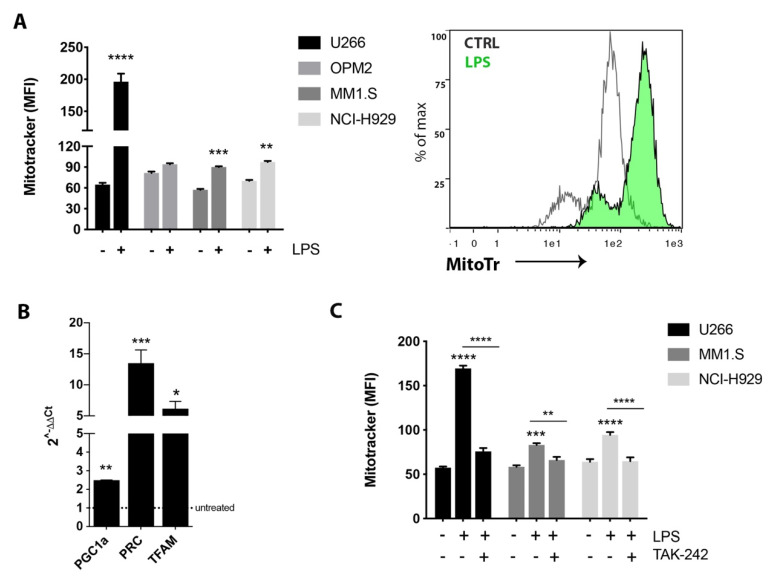
Toll-like receptor 4 (TLR4) activation increased mitochondrial biogenesis in MM cell lines. (**A**) Flow cytometric analysis of Mitotracker Red CMXRos staining after treatment with 2 μg/mL lipopolysaccharide (LPS) for 24 h. A representative flow cytometry histogram shows the comparison of Mitotracker red fluorescence of control vs. LPS treated in U266 cell line. (**B**) Gene expression analysis of mitochondrial biogenesis markers in U266 cells after 6h LPS treatment. B2M gene was used as housekeeping gene. Calculated value of 2^−ΔΔCt in untreated cells was 1. (**C**) Flow cytometric analysis of Mitotracker Red CMXRos staining after treatment with LPS alone or in combination with 10 µM TAK- for 24 h. Bars indicate the standard error means. * *p* < 0.05; ** *p* < 0.01; *** *p* < 0.001; **** *p* < 0.0001.

To investigate whether TLR4 may be involved in the mitochondria biogenesis of human myeloma cell lines (HMCLs), we evaluated the effect of the activation of this pathway on mitochondrial mass. After incubation with LPS for 24 h, HMCLs showed higher mitochondrial mass, as measured by MitoTracker staining using flow cytometric analysis (Figure 1A). Consistent with these data, LPS also significantly enhanced expression of mitochondrial biogenesis markers, such as PGC1α (Peroxisome Proliferator-Activated Receptor Gamma Coactivator 1 alpha), PRC (PGC-1-related coactivator) and TFAM (transcription factor A mitochondrial) (Figure 1B). To further confirm the involvement of TLR4 in mitochondrial biogenesis, U266, MM1.S and NCI-H929 cell lines were treated with the selective TLR4 inhibitor, TAK-242, 2 h before exposure to LPS. Compared to LPS alone, TAK-242/LPS treated cells did not show an increase in mitochondrial mass (Figure 1C).

### 2.2. BTZ Treatment Led to Activation of TLR4 Signaling

**Figure 2:**
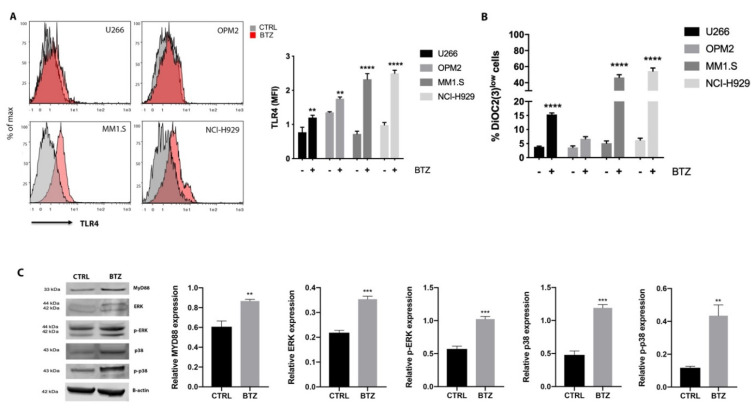
Bortezomib (BTZ) treatment activated TLR4 signaling. (**A**) Flow cytometric analysis for TLR4 expression of multiple myeloma (MM) cell lines after exposure to BTZ for 24 h. Representative histograms of analyzed human myeloma cell lines (HMCLs) are shown. (**B**) Mitochondrial membrane potential was assessed using DiOC2(3) staining following 24 h incubation of HMCLs with BTZ. (**C**) Western blot showing the expression of MyD88, p-ERK, p-p38 and total ERK and p38 in U266 cells after treatment with 15 nM BTZ for 24 h. β-actin protein was used as total protein loading reference. For analysis of Western blot, the optical density of the bands was measured using Scion Image software. All results shown represent the means of four independent experiments; bars indicate the standard error means. ** *p* < 0.01; *** *p* < 0.001; **** *p* < 0.0001.

As the proteasome inhibitor BTZ affects the mitochondria promoting generation of reactive oxygen species (ROS), alteration in the mitochondrial membrane potential and release of cytochrome c [29], we hypothesized that MM cells may activate TLR4 signalling during exposure to BTZ as a stress-responsive mechanism to protect mitochondria against drug-induced apoptosis. Compared to untreated cells, HMCLs treated with BTZ for 24 h increased TLR4 surface expression as evaluated by flow cytometry (Figure 2A). Notably, higher TLR4 expression was measured in MM cell lines with higher BTZ-induced mitochondrial depolarization (MM1.S and NCI-H929, which increased the percentage of depolarized cells by about 41.3% and 49% respectively) (Figure 2B). The activation of the TLR4 pathway mediated by BTZ was then evaluated in U266 cells. Levels of MyD88, ERK, p-ERK, p38 and p-p38 were significantly increased 24 h after BTZ exposure (Figure 2C).

### 2.3. TLR4 Inhibition Restores Pharmacological Response of U266-R to BTZ-Induced Apoptosis

**Figure 3:**
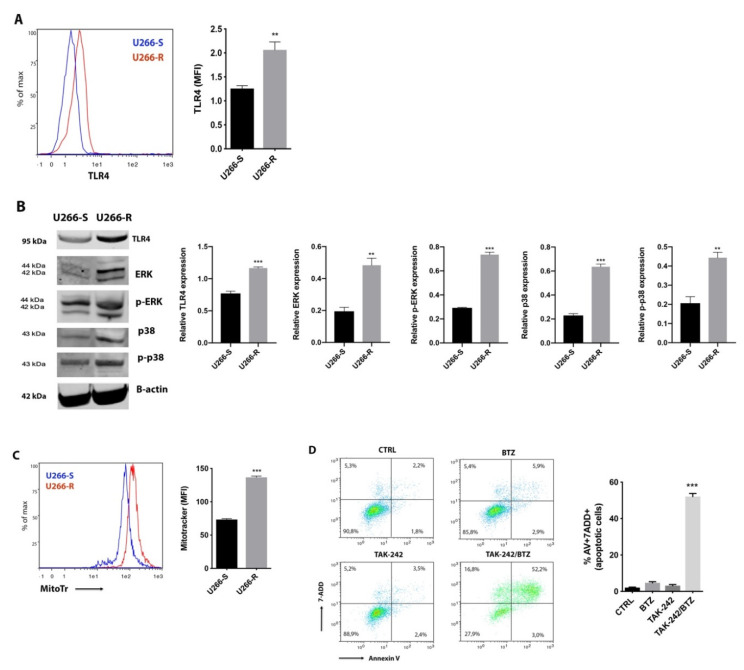
Combined treatment with TLR4 inhibitor and BTZ overcame proteasome inhibitors (PI) resistance and induced apoptosis in U266-R. (**A**) A representative histogram of a flow cytometric analysis showing U266-S (blue line) and U266-R (red line) stained with anti-TLR4 antibody. The TLR4-mean fluorescence intensity (MFI) was calculated and plotted. (**B**) Western blot analysis of TLR4, ERK, p-ERK, p38 and p-p38 in U266-S vs. U266-R. β-actin protein was used as total protein loading reference. For analysis of Western blot, the optical density of the bands was measured using Scion Image software. (**C**) Mitochondrial mass was analyzed using Mitotracker staining. A representative histogram for U266-S (blue line) and U266-R (red line) is shown and Mitotracker-MFI analysis is plotted. (**D**) Representative dot plots of the effect of TAK-242/BTZ treatment on the viability of U266-R cells is shown (left panel). The graph (right panel) shows the mean values of the percentage of apoptotic cells after annexin-V-FITC and 7-ADD staining. All results shown represent the means of four independent experiments; bars indicate the standard error means. ** *p* < 0.01; *** *p* < 0.001.

To better investigate whether TLR4 signalling could be a protective mechanism against loss of mitochondrial membrane potential during BTZ-induced apoptosis, we analyzed TLR4 in U266-R. Compared to BTZ-sensitive cells (U266-S), U266-R showed higher surface expression of TLR4 (Figure 3A), increased protein levels and higher p-38, p-p38, ERK and p-ERK (Figure 3B), indicating a hyperactivation of TLR4 in the BTZ resistant cell line. Moreover, fluorescence values, using Mitotracker staining to evaluate mitochondrial mass, were 136.6 ± 3.4 in U266-R and 73.3 ± 4.7 in U266-S (*p* < 0.001, Figure 3C). Considering that mitochondrial biogenesis is favoured by TLR4 signalling and PI resistance is accompanied by increased reliance on mitochondrial respiration [26], we tested to see if a combination of BTZ with the TLR4 inhibitor TAK-242 might resensitize resistant cells to BTZ-induced apoptosis. U266-R were therefore treated with 15 nM BTZ, 20 μM TAK-242 or their combination. Under this last condition BTZ was added 2 h after TAK-242 exposure. Combinatorial treatment significantly induced cell apoptosis (52% compared to untreated cells; *p* < 0.001, Figure 3D). No toxicity was observed with TAK-242 alone.

### 2.4. Combined Treatment with TLR4 Inhibitor and BTZ Induced Oxidative Stress and Mitochondrial Depolarization Damaging Mitochondrial Dynamics and Energy Metabolism

**Figure 4:**
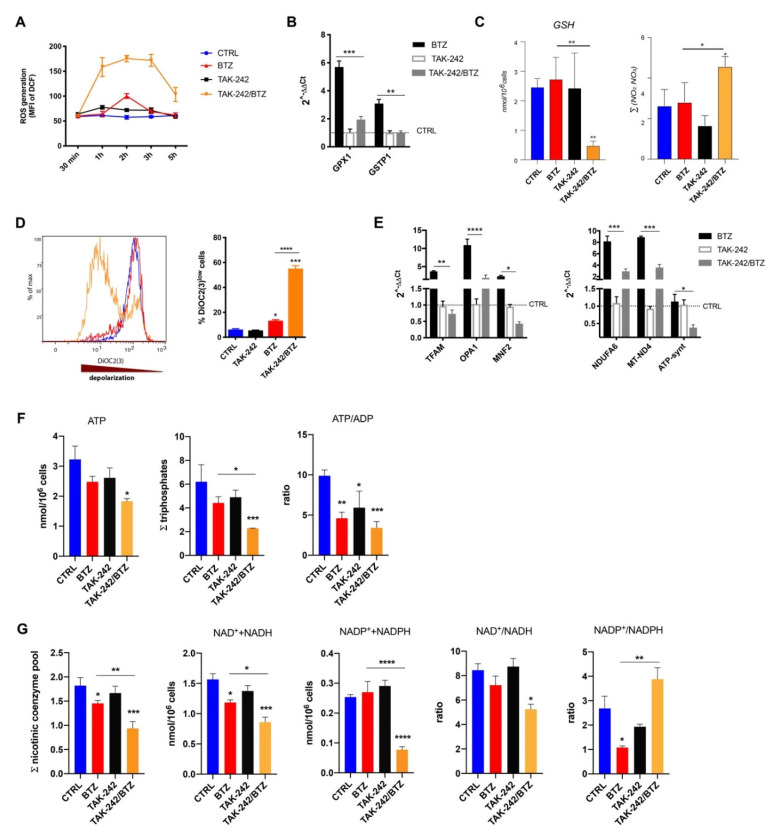
TAK-242/BTZ treatment induced oxidative stress and impaired mitochondria fitness. (**A**) Reactive oxygen species production during drug treatment was measured by oxidation of DCF using flow cytometry. (**B**) The relative gene expression of key cellular antioxidant genes GPX1 and GSTK1 (relative to B2M) was determined by RTqPCR after 4 h. Calculated value of 2^−ΔΔCt^ in untreated cells was 1. (**C**) Levels of GSH and the sum of nitrite + nitrate were determined by HPLC analysis in deproteinized cells after 24 h. (**D**) Mitochondrial membrane potential was assessed following 24 h incubation of U266-R cells with drugs. A representative histogram of a flow cytometric analysis of DiOC2(3) staining (left panel) is shown. (**E**) The mRNA transcription of TFAM, OPA1, MNF2 and NDUFA6, MT-ND4 and ATP-synthase (relative to B2M) was determined by RTqPCR after 4 h. The calculated value of 2^−ΔΔCt^ in untreated cells was 1. (**F**, **G**) ATP, ADP, AMP, NAD^+^, NADH, NADP^+^ and NADPH concentrations were calculated by HPLC analysis in deproteinized cells 24 h post-treatment with BTZ, TAK-242 or their combination. All results shown are mean ± SEM of n ≥ 3 independent experiments; * *p* < 0.05; ** *p* < 0.01; *** *p* < 0.001; **** *p* < 0.0001. (*p* values refer to control or BTZ-treated cells).

We next investigated the link between mitochondrial status, oxidative/nitrosative stress, energy metabolite levels and responsiveness to BTZ in U266-R after treatment with BTZ alone, TAK-242 or their combination. Taking into account that oxidative/nitrosative stress is an important mechanism of PI cytotoxicity, we first evaluated ROS production by using flow cytometry. ROS levels increased only after 2 h exposure to BTZ alone and returned to normal levels within 3 h from treatment (Figure 4A). Normalization of ROS production coincided with the stimulation of antioxidant defenses, as evidenced by increased expression of genes encoding antioxidant enzymes GPX1 (glutathione peroxidase 1) and GSTK1 (glutathione S-Transferase Pi 1, Figure 4B). In contrast, TAK-242/BTZ induced higher and sustained ROS levels and inhibited the upregulation of BTZ-induced antioxidant genes (Figure 4A,B). In order to analyze the multivariate effect of metabolite levels on U266-R treated either with BTZ and/or TAK-242, we performed a principal component analysis (PCA) on metabolite levels integrating all 40 screened metabolites to generate principal components (PCs, Figure S1). We found that among the highest contributors to PCA, Glutathione (GSH) and nitrite were highly represented in PC1 with a contribution of 0.50 and 0.81, respectively, and also nitrate contribution was also relatively high, with a contribution to PC1 and PC2 equal to 0.51 (Figure S1 and Table S1). Indeed, the TAK-242/BTZ combination produced a dramatic 80% decrease in GSH levels (*p* < 0.001 compared to both control and BTZ or TAK-242 alone). The direct quantification by HPLC of the stable end products of nitric oxide formation, showed almost twice the values in the sum of nitrite + nitrate in cells treated with TAK-242/BTZ (*p* < 0.001 compared to both control and BTZ or TAK-242 alone), thus indicating the dangerous coincidence of oxidative and nitrosative stress (Figure 4C). Elevated ROS and RNS (reactive nitrogen species) impaired mitochondrial membrane potential (Figure 4D). Analyzing the effect of each treatment on mitochondrial polarization status, we noted that BTZ treated cells had about 6.7% of depolarized cells compared to the control (*p* < 0.05). On the other hand, TAK-242/BTZ treatment induced mitochondrial depolarization of about 59.3% (*p* < 0.001), indicating that drug combination led to the leakage of protons across the inner mitochondrial membrane.

To respond to mitochondrial stress, cells treated with BTZ alone promote a compensatory upregulation of the OXPHOS-related genes NDUFA-6 (NADH:ubiquinone oxidoreductase subunit A6), MT-ND4 (Mitochondrially Encoded NADH:Ubiquinone Oxidoreductase Core Subunit 4), as well as OPA1 (Optic dominant atrophy 1) and MFN2 (Mitofusin 2) associated with mitochondrial fusion and TFAM, as a regulator of mitochondrial biogenesis (Figure 4E). On the contrary, TAK-242/BTZ almost abolished the BTZ-induced upregulation of OXPHOS-, mitochondrial biogenesis- and fusion-related genes, suggesting a close link with TAK-242/BTZ-mediated mitochondrial impairment. Notably, even if ATP, ADP, AMP, GTP, UTP and CTP were not relatively high contributors to PC1, they collectively explain the 5% of total variances between groups (Table S1). In agreement with these data, we observed that U266-R treated with TAK-242/BTZ had significantly lower concentrations of both ATP and a sum of the triphosphate nucleosides (ATP+ GTP+UTP+CTP) than those measured in cells treated with BTZ alone (−27% and −49%, respectively, *p* < 0.05 and *p* < 0.01, Figure 4F). This energy crisis was possibly mediated by mitochondrial malfunctioning, as indicated by the lower values of the ATP/ADP ratio observed in U266-R treated with the drug combination (−31% compared to BTZ alone, *p* < 0.05; Figure 4F), suggesting a decrease in the mitochondrial phosphorylating capacity. Thanks to the sample processing and HPLC method used, preserving the redox state of easily oxidizable substrates and allowing separation and exact quantification of about 40 metabolites of biochemical relevance, we found that the sum of the nicotinic coenzyme pool (NAD^+^ + NADH + NADP^+^ + NADPH) in control, BTZ-, TAK-242- and TAK-242/BTZ-treated cells was, respectively, 1.82 ± 0.17, 1.45 ± 0.06, 1.67 ± 0.14 and 0.94 ± 0.14 nmol/10^6^ cells (drug combination vs. BTZ alone: *p* < 0.01, Figure 4G). When considered separately NAD^+^ + NADH and NADP^+^ + NADPH, no differences were observed comparing the values in control (1.57 ± 0.17 and 0.254 ± 0.009 nmol/10^6^ cells) and cells exposed to TAK-242 (1.37 ± 0.48 and 0.29 ± 0.019 nmol/10^6^ cells, Figure 4G). Conversely, BTZ-treated cells had lower values of NAD^+^ + NADH (1.18 ± 0.08, *p* < 0.05 compared to control) and normal values of NADP^+^ + NADPH (0.270 ± 0.026 nmol/10^6^ cells), whilst cells exposed to TAK-242/BTZ showed significant depletion of both NAD^+^ + NADH (0.814 ± 0.152, *p* < 0.05 compared to BTZ alone) and NADP^+^ + NADPH (0.078 ± 0.009 nmol/10^6^ cells, *p* < 0.001 compared to BTZ treated cells). The NAD^+^/NADH ratio was 8.45 ± 0.92, 7.22 ± 1.30 and 8.74 ± 1.16, respectively, in control, BTZ- and TAK-242-treated cells and decreased to 5.25 ± 0.73 in cells exposed to BTZ/TAK-242 (*p* < 0.05 compared to control, Figure 4G), indicating activation of glycolysis only when treating cells with the drug combination. Interestingly, the NADP^+^/NADPH ratio was 2.68 ± 0.87 in control, 1.08 ± 0.12 in cells treated with BTZ (*p* < 0.05 compared to control), 1.93 ± 0.18 in cells exposed to TAK-242 (not significantly different) and 3.88 ± 0.81 in TAK-2427/BTZ treated cells (*p* < 0.01, compared to BTZ treatment alone, Figure 4G). Such observations were confirmed by PCA, in which NADP^+^ and NADPH were among the highest contributors to discriminate the TAK-242/BTZ group from the other groups (Figure S1 and Table S1). Taken together our evidence suggests that U266-R cells responded to BTZ by increasing the reduction of NADP^+^ to NADPH, presumably to maintaining high GSH concentrations for counteracting oxidative/nitrosative stress. Conversely, cells responded to TAK-242/BTZ by increasing the oxidation of NADPH to NADP^+^, possibly through the involvement of NADPH-oxidase activation during oxidative stress.

### 2.5. Combination of TAK-242/BTZ Decreased the Hexosamine Biosynthetic Pathway Products

**Figure 5:**
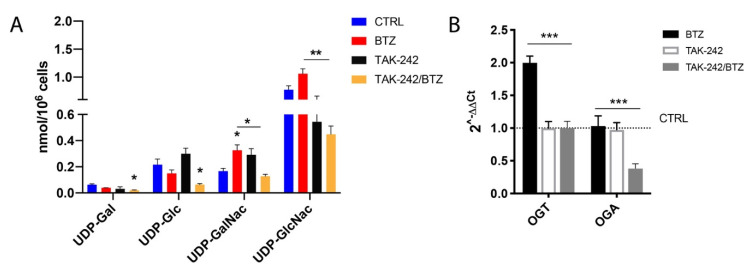
TAK-242/BTZ treatment impaired the hexosamine biosynthetic pathway. (**A**) Concentrations of glycosylated UDP-derivatives were calculated by HPLC analysis in deproteinized cells 24 h post-treatment with BTZ, TAK-242 or their combination. Values are the mean of three different experiments and are expressed as nmol/10^6^ cells. *UDP-Gal = UDP-galactose; UDP-Glc = UDP-glucose; UDP-GalNac = UDP-N-acetylgalactosamine; UDP-GlcNac = UDP-N-acetylglucosamine.* (**B**) The mRNA transcription of OGT and OGA (relative to B2M) was determined by RTqPCR after 24 h. Calculated value of 2^−ΔΔCt in untreated cells was 1. * *p* < 0.05; ** *p* < 0.01; *** *p* < 0.001 (*p* values refer to control or BTZ-treated cells).

Our unpublished data suggest that U266-R cells have the marked signature of high activity of the HBP, necessary for efficient protein glycosylation, as evidenced by the high levels of the main compounds (UDP-Gal, UDP-Gluc, UDP-GalNac, UDP-GlucNac) involved in this post-translational protein modification. Such a hypothesis was strongly supported by PCA on metabolite levels, highlighting UDP-Gal (squared cosine of contribution to PC1 = 0.56), UDP-Gluc (squared cosine of contribution to PC1 = 0.59), UDP-GalNac (squared cosine of contribution to PC1 = 0.51) and UDP-GlucNac (squared cosine of contribution to PC1 = 0.70) as relatively high contributors to group clustering (Figure S1 and Table S1). Figure 5 illustrates the effect of the TAK-242/BTZ combination on HBP. The proteasome reduced UDP-Gal levels of about 38.4% (*p* < 0.05 compared to control) and increased UDP-GalNac (from 0.17 ± 0.03 to 0.32 ± 0.07; *p* < 0.05) (Figure 5A). TAK-242 did not cause significant changes of these UDP-derivatives, whilst the combination of TAK-242/BTZ produced a dramatic decrease of their concentrations. In particular, the two compounds necessary for N-glycosylation (UDP-GalNac) and O-glycosylation (UDP-GlcNac) of proteins underwent, respectively, a 61% and 58% decrease (*p* < 0.01 compared to BTZ alone; Figure 5A) indicating a remarkable loss of efficiency in this post-translational protein modification. To confirm these observations, we next explored the expression of genes encoding O-GlcNAc transferase (OGT) and O-GlcNAcase (OGA) enzymes, responsible, respectively, for the production and removal of the O-GlcNAc [30]. We found that the TAK-242/BTZ combination significantly downregulated OGA compared to BTZ treatment alone and abolished the BTZ-induced OGT upregulation (*p* < 0.001; Figure 5B).

### 2.6. Mitochondrial Impairment Induced by TAK-242/BTZ Cotreatment Increased Mitophagy

**Figure 6:**
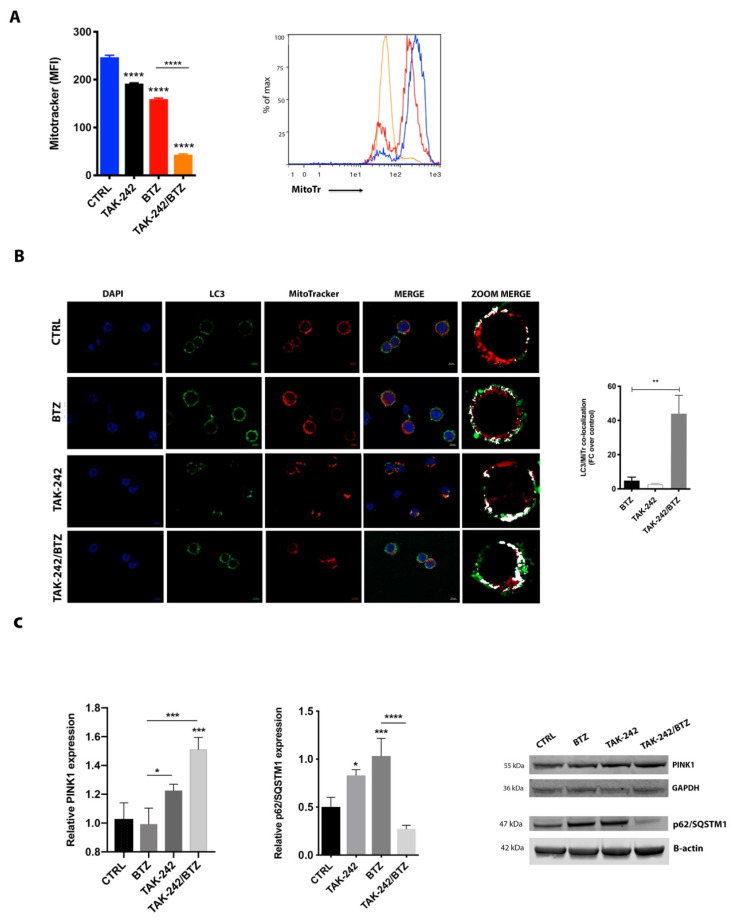
The combination of TAK-242/BTZ activated mitophagy. (**A**) Flow cytometric analysis of U266-R for mitochondrial mass 24 h post-treatment as determined by MitoTracker Red fluorescence. A representative histogram of MitoTracker staining (right panel) is shown. (**B**) Immunofluorescence for the colocalization of LC3 (green) and MitoTracker (red) in U266-R after 24 h treatment with BTZ and its combination with TLR-4 inhibitor. Counterstaining of cells was performed by using the nuclear dye, DAPI (blue). Quantitative analysis of the colocalization of LC3 with MitoTracker was plotted (right panel). FC = fold changes. Scale bar: 10 μm (**C**) Analysis of PINK1 and p62/SQSTM1 protein expression after drug treatments for 24 h. β-actin or GAPDH proteins were used as total protein loading reference. For analysis of Western blot, the optical density of the bands was measured using Scion Image software (https://imagej.net/Fiji). All showed results represent the means of four independent experiments; error bars denote SD. * *p* < 0.05; ** *p* < 0.01; *** *p* < 0.001; **** *p* < 0.0001 (*p* values refer to control or BTZ-treated cells).

Increased oxidative stress is a feature of mitochondrial dysfunction, which has been found to activate mitophagy and target mitochondria for degradation [31]. Consistent with this, in TAK-242/BTZ treated cells, the strong depolarization and energetic impairment of mitochondria were accompanied by a drastic reduction of mitochondrial mass (Figure 6A). Indeed, while BTZ treatment reduced the MitoTracker-MFI value from 244 ± 9.5 (control) to 168 ± 11.3 (*p* < 0.001), cells treated with TAK-242/BTZ exhibited a MitoTracker-MFI value of 42 ± 2.8 (*p* < 0.001).

We next evaluated if the effects of these changes in overall mitochondrial mass were linked to mitophagy, a fundamental process of the mitochondrial quality control aimed at the removal of dysfunctional mitochondria. By evaluating mitophagy using the colocalization of Mitotracker stained mitochondria with LC3 protein (a constituent of the autophagosome), we found that BTZ treatment induced a moderate increase in mitophagy (Figure 6B). However, a great activation of mitophagy was observed in U266-R cells following exposure to TAK-242/BTZ (about 38-fold higher than BTZ treated cells; *p* < 0.001), thus suggesting that mitochondrial impairment induced by the drug combination led to the activation of mitophagy finalized to apoptosis, rather than to protect cells from the deleterious activity of defective mitochondria. In accordance with these results, treatment with TAK-242/BTZ significantly increased expression of PINK1 (PTEN-induced kinase 1) (*p* < 0.05 compared to control; Figure 6C). Moreover, investigating the expression of p62/SQSTM1, which regulates mitochondrial clustering during mitophagy [32,33], we observed that both BTZ and TAK-242 alone increased p62/SQSTM1 expression, whilst their combination significantly downregulated the protein level compared to BTZ alone (*p* < 0.001).

### 2.7. Targeting TLR4 and Proteasome Activities Resensitized CD138+ Cells from Refractory MM Patients

**Figure 7:**
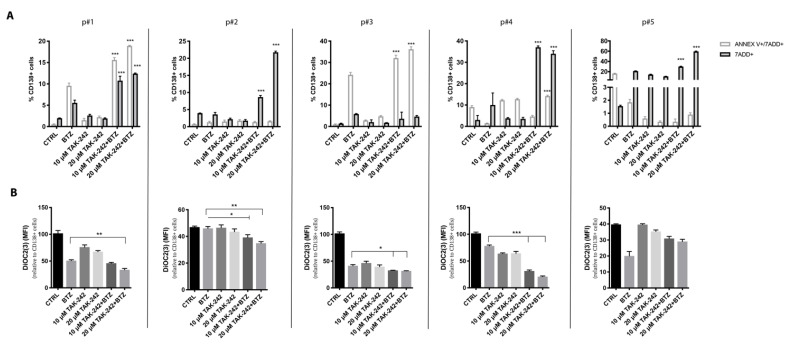
Targeting TLR4 signaling and proteasome activities resensitized to BTZ myeloma PCs cells from refractory patients. CD138+ cells obtained from refractory MM patients were treated with BTZ (5 nM) in combination or not with TAK-242 (10–20 µM). (**A**) The percentage of CD138+/annexin V+/7-ADD+ or CD138+/7ADD+ cells were estimated by flow cytometry 48 h post-treatment. (**B**) Mitochondrial membrane potential was assessed 24 h post-treatment analyzing MFI of DiOC2(3) staining on CD138+ cells by using flow cytometry. Bars indicate the standard error means. * *p* < 0.05; ** *p* < 0.01; *** *p* < 0.001.

To evaluate whether TLR4 inhibition resensitizes primary cells to BTZ-induced apoptosis, CD138+ cells derived from five refractory MM patients were treated for 48h with 5 nM BTZ, 10–20 μM TAK-242 or their combination. TAK-242 alone or in combination with BTZ did never show cytotoxicity toward CD138- cells (Figure S2). Treatment with both drugs significantly decreased the viability of CD138+ cells (Figure 7A). To confirm that TAK-242/BTZ damages mitochondria causing dysfunction of cell metabolism and apoptosis, we evaluated the mitochondrial polarization status in CD138+ cells 24 h post-treatment. In all samples but p#5, the combination of BTZ with TLR4 inhibitor induced higher mitochondrial depolarization than that caused by BTZ alone (Figure 7B). It is worth underlining that p#5 corresponds to the sample showing the lowest TAK-242/BTZ cytotoxicity. Taken together, these data demonstrate that TLR4 inhibition significantly resensitized CD138+ cells from a small group of MM patients refractory to BTZ thanks to the induction of remarkable mitochondrial depolarization associated with cell death.

## 3. Discussion

Even though PI and immunomodulatory drugs (IMiDs) have led to substantial outcome improvements in MM patients, the development of novel strategies of drug combinations is needed to overcome resistance. The current study identifies TLR4 signaling as a contributor pathway to PI resistance. TLR4 belongs to the family of pattern recognition receptors (PRRs) that recognize PAMPs, also binding endogenous molecules inducing a proinflammatory response. TLR4-mediated inflammation is involved in several cancers and also in chronic diseases, having a pivotal role as amplifier of the inflammatory response [9,34,35,36]. Previous studies have implicated the TLR4 pathway in MM cell proliferation and immune escape mechanisms [15,16], as well as in protumor activation of MM-MSC [17]. Our study suggests that activation of TLR4 signaling in MM cells promotes mitochondrial biogenesis and favours the acquisition of resistance to BTZ. In particular, we found that TLR4 activation by LPS stimulation increases MM cell mitochondrial mass and enhances expression of PGC1α (the master regulator of mitogenesis), PRC (a transcriptional cofactor that activates genes involved in mitochondrial respiratory function), and TFAM (a key regulator of mitochondrial biogenesis that promotes mtDNA replication and transcription). To further confirm that TLR4 activation induced mitochondrial biogenesis in HMCLs, we used TAK-242 as the selective TLR4 inhibitor that binds to Cys747 in the intracellular domain of receptor and disrupts the interaction of TLR4 with its adaptor molecules [37]. As expected, LPS treatment was not able to induce mitochondrial mass after TLR4 inhibition.

MM cells are particularly sensitive to increased ER stress because they constitutively express ER stress survival factors for proper antibody assembly and secretion, thus ensuring their secretory cell function [21]. The UPR, a signaling activated by the accumulation of unfolded protein within ER, attempts to reduce the protein load on the ER and increase its folding capacity. However, unresolved ER stress results in the activation of apoptosis with the transition of the UPR from a protective to an apoptotic response. MM cells are inherently sensitive to PI because these drugs activate UPR and ER stress-induced apoptosis [19]. It has been recently demonstrated that the TLR4 pathway provides a protective effect against BTZ-induced ER stress and pretreatment of MM cells with LPS significantly reduces BTZ-induced apoptosis [22]. The close contact between ER and mitochondria significantly impacts mitochondrial dynamics (fusion, fission and mitophagy) [38] and supports communication between these two organelles. Synthesis and transfer of lipids, exchange of Ca^2+^ regulating ER chaperones, mitochondrial ATP production and apoptosis are the main processes influenced by the ER-mitochondria contacts [39]. Hence, stressing the ER triggers the intrinsic pathway of apoptosis through mitochondrial depolarization, cytochrome c release and its downstream caspase-9 activation. Here, we demonstrate that MM cells increase TLR4 expression and signalling after exposure to BTZ as a stress-responsive mechanism to counteract BTZ-induced mitochondrial depolarization. To support this hypothesis, it is important to note that U266-R showed constitutively increased TLR4 expression and signalling, as well as higher values of mitochondrial mass. Additionally, we previously found that U266 cells resistant to BTZ had higher values of parameters reflecting mitochondrial function (membrane potential, ATP/ADP ratio, NAD^+^/NADH ratio) than those measured in U266 sensitive to drugs, thereby confirming that the PI-resistant phenotype is accompanied by an increase in mitochondrial biomass and increased reliance on mitochondrial respiration [26,28,40,41]. Thus, since TLR4 regulates mitochondrial biogenesis and BTZ-resistant cells rely on mitochondrial fitness, a link among TLR4 upregulation and PI resistance may exist. To demonstrate this link, we used TAK-242 to selectively inhibit TLR4 signaling. The combination of TAK-242 with BTZ strongly resensitized U266-R to PI through the generation of higher and extended oxidative stress, strong mitochondrial depolarization, severe impairment of mitochondrial respiration, pronounced energy crisis and inhibition of UDP-derivatives-dependent protein glycosylation which in turn activated mitophagy and cell apoptosis. Indeed, BTZ alone provoked a transitory (3 h) increase in ROS levels and a steady upregulation of the genes encoding antioxidant enzymes such as GPX1 and GSTK1, with consequent modest mitochondrial depolarization. The TAK-242/BTZ combination activated a strong pro-oxidant status increasing ROS and RNS, decreasing GSH levels and the expression of antioxidant genes. High levels of oxidative stress lead to apoptosis through various mechanisms, including UPR induction, which reinforces oxidative stress, as well as permeabilization of the outer mitochondrial membrane and cytochrome c release [42]. Consistent with these findings, cells exposed to the TAK-242/BTZ combination displayed impaired mitochondrial respiration with a marked imbalance in energy metabolism. Particularly, we found that the combination of BTZ with TAK-242, besides decreasing ATP and the sum of triphosphate nucleosides (ATP+GTP+UTP+CTP), caused a reduction in the sum of NAD^+^+NADH with a decrease in their ratio (NAD^+^/NADH). This implies that the drug combination had a double negative effect on these nicotinic coenzymes: on one side, it provoked a net decrease in their concentration; on the other side, it caused a significant change in their respective oxidoreductive states. Whilst the first effect should have occurred through the increase in ADP-ribosylation reactions mediated by the activation of NAD^+^-glycohydrolase and PARP [43], the second effect should have been due to a decreased efficiency in the mitochondrial electron transport chain (ETC) with consequent acceleration of the glycolytic rate to ensure adequate ATP (and triphosphate nucleosides) supply for the cell energy demand. The cumulative effect of the two phenomena is the imbalance between energy production and consumption with dramatic impoverishment of high-energy phosphate levels and energy crisis. The decrease in the triphosphate nucleosides, particularly UTP, is crucial to ensure adequate concentrations of UDP-derivatives (UDP-Gal, UDP-Glc, UDP-GalNac, UDP-GlcNac) involved in HBP and O-linked and N-linked glycosylation of proteins. The cumulative effects of ER stress and decreased efficiency of protein glycosylation, caused by the TAK-242/BTZ combination, might represent fundamental target rescuing sensitivity to BTZ and tremendously increased cytotoxicity. ATP-synthase expression was downregulated as well as the BTZ-upregulated OXPHOS-related genes (NDUFA6 and MT-ND4). TAK-242/BTZ also markedly affected the concentrations of NADP^+^+NADPH and the NADP^+^/NADPH ratio. The decreased availability of NADPH is essential for promoting the regeneration of GSH from GSSG through the action of NADPH-dependent glutathione reductase. GSH is essential, in turn, for the activity of several antioxidant enzymes including GPX and glutathione S-transferase [44], whose genes were downregulated in cells after TAK-242/BTZ exposure. Thus, we can speculate that the enhancement of oxidative stress triggers a “vicious cycle” involving ROS generated by mitochondrial malfunctioning (inability to manage the electron flow for the tetravalent reduction of molecular oxygen through ETC), which in turn generates additional ROS leading to further damage to biomolecules and apoptosis.

Mitochondria respond to environmental changes and energy demands of the cell by fusing together to form an interconnected reticulum, and by dividing to either increase mitochondrial number or to segregate portions of the organelle for degradation via mitophagy [45]. The interplay between mitochondrial dynamics (repetitive cycles of fusion and fission) and mitophagy assures cell homeostasis. To protect them from mitochondrial dysfunction, BTZ-treated cells activated mitochondrial fusion and biogenesis, as demonstrated by the upregulation of mitofusin, OPA1 and TFAM. Mitochondrial fusion prevents removal of impaired mitochondria through the accumulation of the mitofusin protein that allows them to reengage in the fusion process despite their deprived energetic state so improving mitochondrial activity. Mitochondria can fuse only if their activity is above a certain threshold: depolarization below a certain ΔΨ_m_ is a prerequisite for mitophagy which has a role at the end of the axis of quality control of mitochondria [46]. Therefore, the low levels of depolarized mitochondria allow BTZ-treated cells to coordinate mitochondrial fusion, biogenesis and mitophagy, leading to the maintenance of mitochondrial fitness. Additionally, since OPA1 is implicated in mitochondrial cristae remodelling and regulation of ETC-linked OXPHOS, its increase in resistant cells certainly impacts mitochondrial energy production and contributes to allow high oxidative metabolism and aerobic ATP generation. In contrast, U266-R lost the capacity to activate mitochondrial fusion and biogenesis after TAK-242/BTZ exposure significantly decreased mitofusin and OPA1, as well as TFAM expression. Therefore, in these treated cells apoptosis upon mitochondrial dysfunction is closely related to a strong mitophagy response which facilitates cytochrome c release from mitochondria. TAK-242/BTZ treatment increased expression of PINK1, which accumulates at the outer mitochondrial membrane (OMM) and recruits Parkin to initiate mitophagy [47]. Generally, mitochondrial fragmentation precedes mitophagy as mitochondria that are smaller and easier to be engulfed by the autophagosomes [48]. During mitophagy, there is a robust mitochondrial translocation of p62/SQSTM1 which usually binds and targets ubiquitinated proteins to autophagic degradation. Accumulating evidence supports that p62 protects cells upon mitochondrial depolarization by its ability to promote mitochondrial aggregation [33,49]. We found that on the contrary, BTZ and TAK-242 alone increased p62/SQSTM1 expression, their combination significantly downregulated the protein level, suggesting that TAK-242/BTZ abrogates the protective role of mitochondrial clustering that favour the retention of cytochrome c which in turn defends cells against apoptosis.

Finally, we demonstrated the efficacy of the TAK-242/BTZ combination in primary human MM cells from refractory patients. Inhibition of TLR4 increased BTZ-induced mitochondrial depolarization, resensitizing resistant CD138+ cells to PI. Importantly, the combination did not exhibit cytotoxicity toward CD138^−^ cells.

## 4. Material and Methods

### 4.1. MM Cell Lines and CD138^+^ Plasma Cells

HMCLs were purchased from ATCC (Manassas, VA, USA). Cells were grown in Roswell Park Memorial Institute (RPMI) 1640 medium supplemented with 10% (for MM1.S, OPM2, NCI-H929, all tested for mycoplasma free) or 20% (for U266) fetal bovine serum and 1% penicillin-streptomycin. All HMCLs were screened for TLR4 expression using flow cytometry (Figure S3). U266-R cells were selected for BTZ resistance by exposure to progressively higher concentrations of BTZ and reached resistance to higher doses of the drug (EC50 value:100 nM) after a quiescent state for a period of about 1 month. Bone marrow from 5 refractory MM patients were collected following written informed consent (Azienda ospedaliero Universitaria Policlinico-Vittorio Emanuele, #34/2013/VE). Mononuclear cells were obtained by gradient centrifugation on Ficoll (Biochrom, Berlin, Germany). Cells were purified using CD138 microbeads according to the manufacturer’s instructions (Miltenyi Biotech, Bologna, Italy), tested for purity using flow cytometry and then used for in vitro experiments. Clinical data of patients included in this study are provided in Table 1.

Cells were treated with 2 μg/mL LPS (Sigma-Aldrich, Mylan, Italy) to activate TLR4. The TLR4 inhibitor TAK-242 was purchased from Sigma-Aldrich. Commercially available BTZ was used.

### 4.2. Flow Cytometry

TLR4 expression after BTZ stimulation was evaluated by flow cytometry using antihuman CD284-APC antibody (130-096-236; clone HTA125; Miltenyi Biotech) on a MACSQuant Analyzer (Miltenyi Biotech) according to the manufacturer’s instructions.

The levels of reactive oxygen species (ROS) were detected using the 2′,7′-Dichlorodihydrofluorescein acetate (Sigma-Aldrich) and fluorescence intensity was measured according to the fluorescence detection conditions of FITC.

A membrane potential probe, the 3,3′-Diethyloxacarbocyanine Iodide (DiOC2(3)), was used to evaluate the mitochondrial membrane potential. Cells were incubated with 10 μM DiOC2(3) (Thermo Fisher Scientific, Milan, Italy) for 30 min at 37 °C, washed twice, resuspended in PBS and analyzed by flow cytometry through the detection of the green fluorescence intensity of DiOC2(3). To evaluate mitochondrial depolarization in CD138+ cells from MM patients, DiOC2(3) labeled cells were then incubated with APC antihuman CD138 monoclonal antibody (clone B-A38; Beckman Coulter, Mylan, Italy) at room temperature for 15 min. After centrifugation, cells were resuspended in PBS and then analyzed using flow cytometry.

In order to measure changes in the mitochondrial mass, cells were reacted with 200 nM MitoTracker Red CMXRos probe (Thermo Fisher Scientific, Milan, Italy) for 30 min at 37 °C, according to the manufacturer’s instructions. After being washed twice, labelled mitochondria were analyzed by flow cytometry.

To evaluate apoptosis after drug treatment, cells were stained with annexin AV FITC/7-ADD assay kit (Beckman Coulter) according to the manufacturer’s instructions. To evaluate apoptosis in CD138+ plasma cells, samples were incubated with APC antihuman CD138 monoclonal antibody at room temperature for 15 min. After centrifugation, cells were resuspended in PBS, stained with annexin AV FITC/7-ADD assay kit. The apoptotic population was immediately evaluated by flow cytometry. The percentages of early apoptotic cells (annexin V^+^/7-ADD^−^) and late apoptotic cells (annexin V^+^/7-ADD^+^) were calculated and graphed.

### 4.3. RT-qPCR

After RNA extraction, reverse transcription was performed by using the High Capacity cDNA Reverse Transcription Kit (Thermo Fisher Scientific). Then the relative transcription of specific genes was determined by RTqPCR using Brilliant III Ultra-Fast SYBR Green QPCR Master Mix (Agilent Technologies, Milan, Italy) and 7900HT Fast Real-Time PCR System (Thermo Fisher. Expression of the following human genes was evaluated:

TFAM (*Fw: CCGAGGTGGTTTTCATCTGT; Rw: AGTCTTCAGCTTTTCCTGCG*); PRC (*Fw:CACTGGTTGACCCTGTTCCT; Rw: GTGTTTCAGGGCTTCTCTGC*); PGC1α (*Fw: ATGAAGGGTACTTTTCTGCCCC; Rw: GGTCTTCACCAACCAGAGCA*); GPX1 (*Fw: CAGTCGGTGTATGCCTTCTCG; Rw: GAGGGACGCCACATTCTCG*); GSTK1 (*Fw: CTGGGCTTCGAGATCCTGTG; Rw: GGCAGACAAACTTCCACTGTC*); OPA1 (*Fw: GTGCTGCCCGCCTAGAAA; Rw: TGACAGGCACCCGTACTCAGT*); MNF2 (*Fw: GCTCGGAGGCACATGAAAGT; Rw: ATCACGGTGCTCTTCCCATT*); NDUFA6 (*Fw: CAAGATGGCGGGGAGCGG; Rw: GTATAGTGAGTTTATTTGTGCTC*); MT-ND4 (*Fw: ACAAGCTCCATCTGCCTACGACAA; Rw: TTATGAGAATGACTGCGCCGGTGA*); ATP-synthase (*Fw: AGCTCAGCTCTTACTGCGG; Rw: GGTGGTAGTCCCTCATCAAACT*); OGA (*Rw: TGCAACTTGCCTACTCATCAC, Fw: TTTCTGGGCCCGTACAAAGG*), OGT (*Rw: ACAGCACAGAACCAACGAAAC -3′, Rw: GCT CAA TTG CCT CCT GCA AC*); B2M (*Fw: AGCAGCATCATGGAGGTTTG; Rw: AGCCCTCCTAGAGCTACCTG*); GAPDH (*Fw: AATGGGCAGCCGTTAGGAAA; Rw: GCCCAATACGACCAAATCAGAG*).

For each sample, the relative expression level of the mRNA of interest was determined by comparison with the control housekeeping genes B2M and GAPDH using the 2^^−ΔΔCt^ method [48].

### 4.4. Western Blot Analysis

Briefly, for Western blot analysis 50 μg of protein was loaded onto a 12% polyacrylamide gel Mini- PROTEAN TGXTM (BIO-RAD, Milan, Italy) followed by electrotransfer to nitrocellulose membrane Trans- Blot TurboTM (BIO-RAD) using Trans-Blot SE Semi-Dry Transfer Cell (BIO-RAD). Subsequently, membrane was blocked in Odyssey Blocking Buffer (Licor, Milan, Italy) for 1h at room temperature. After blocking, the membrane was three times washed in PBS for 5 min and incubated with primary antibodies against human TLR4, MyD88 (sc-293072, sc-11356, Santa Cruz Biotechnology, Santa Cruz, CA, USA), p38, phospho-p38, ERK, phospho-ERK (#9212, #9211, #4377, #9102 Cell Signaling Technology, Danvers, MA, USA), p62/SQSTM1, PINK1, GAPDH and β-actin (ab109012, ab8226, ab23707, ab181602, Abcam, Milan, Italy), overnight at 4 °C. Next day, membranes were three times washed in PBS for 5 min and incubated with Infrared antimouse IRDye800CW (1:5000) and antirabbit IRDye700CW secondary antibodies (1:5000) in PBS/0.5% Tween-20 for 1 h at room temperature. All antibodies were diluted in Odyssey Blocking Buffer. The blots were visualized using Odyssey Infrared Imaging Scanner (Licor, Milan, Italy) and protein levels were quantified by densitometric analysis of antibody responses. Data were normalized to protein levels of β-actin.

### 4.5. HPLC Materials and Method

All ultrapure standards, used for the evaluation of cellular metabolic profile, tetrabutylammonium hydroxide and potassium di-hydrogen phosphate (KH_2_PO_4_) suitable for all buffer preparations, were purchased from Sigma-Aldrich (St. Louis, MO, USA) and diluted in Ultrapure water (18.3 MΩ cm) (Milli-Q Synthesis A10, Millipore, Burlington, MA, USA). Ultrapure solvents (HPLC-grade methanol and chloroform and far-UV acetonitrile) were supplied by J. T. Baker Inc. (Phillipsburgh, NJ, USA).

Metabolic analysis was driven after deproteinization of cell samples according to a protocol suitable to obtain protein-free extracts for further HPLC analysis of acid labile and easily oxidizable compounds [50]. Cells were washed twice with PBS at pH 7.4 and pelleted by centrifugation at 1860× *g* for 5 min at 4 °C. Cell pellets were deproteinized with the addition of 1 mL of ice-cold, nitrogen-saturated, CH_3_CN + 10 mM KH_2_PO_4_, pH 7.4 (3:1, v:v). After vigorous mixing for 60 s, samples were centrifuged at 20,690× *g* for 10 min at 4 °C. The organic solvent was removed from the deproteinizing mixture using two washings with 5 mL of chloroform. The upper aqueous phase obtained by centrifugation at the same conditions, was then used for the HPLC analysis of low molecular weight metabolites. Simultaneous separation of 50 low molecular weight metabolites related to energy metabolism, oxidative/nitrosative stress, antioxidants, and including high energy phosphates (ATP, ADP, AMP, GTP, GDP, GMP, UTP, UDP, UMP, CTP, CDP, CMP), oxidized and reduced nicotinic coenzymes (NAD^+^, NADH, NADP^+^, NADPH), glycosylated UDP-derivatives (UDP-galactose, UDP-glucose, UDP-N-acetyl-glucosamine, UDP-N-acetyl-galactosamine) reduced glutathione (GSH), nitrite and nitrate, was carried out using a Hypersil C-18, 250 × 4.6 mm, 5 µm particle size column, provided with its own guard column (Thermo Fisher Scientific, Rodano, Milan, Italy), following previously established ion pairing HPLC methods [50,51]. The HPLC apparatus was based on a SpectraSYSTEM P4000 pump (Thermo Fisher Scientific, Rodano, Milan, Italy) interfaced to a highly sensitive UV6000LP diode array detector (Thermo Fisher Scientific, Rodano, Milan, Italy), equipped with a 5 cm light path flow cell and set up between 200 and 300 nm wavelength. Assignment and calculations of the aforementioned compounds in cell extracts, were performed by comparing retention times, absorption spectra, and area of the peaks (calculated at 260 nm wavelength for all compounds but GSH, nitrite and nitrate that were calculated at 206 nm wavelength) of chromatographic runs of mixtures containing known concentrations of ultrapure standards.

### 4.6. Immunofluorescence

Following drug treatment, cells were labeled with 200 nM MitoTracker Red CMXRos probe (M7512, Thermo Fisher Scientific, Rodano, Milan, Italy). After being washed twice, labelled cells were adhered to slides by cytospin and subsequently fixed with 4% formaldehyde for 20 min at room temperature, protected from exposure to light. After three washings in PBS for 5 min, cells were fixed using 4% paraformaldehyde, permeabilized using 0.1% Triton X, and blocked to prevent nonspecific antibody binding using 2% bovine serum albumin. The slides were then incubated overnight at 4 °C with the primary antibody against LC3-II-rabbit (L7543, Sigma-Aldrich, Milan, Italy) at 1:100 dilution. Next day, cells were washed three times in PBS for 5 min and incubated with antirabbit FITC secondary antibody at 1:200 dilution for 1h at room temperature. The slides were mounted with medium containing DAPI (4,6-diamidino-2-phenylindole) to visualize nuclei. The fluorescent images were obtained using a Zeiss Axio Imager Z1 Microscope with Apotome 2 system (Zeiss, Milan, Italy).

### 4.7. Statistical Analysis

PCA was performed as previously described [52,53,54]. Briefly, loading individual metabolite levels per sample per group. PCA, explained variances and the quality of the representation of the variables on the factor map expressed as squared cosine, are shown. PCA is expressed as a biplot of variables and key colored arrows representing contributions of variables to PCs. A cutoff equal to 0.50 of squared cosine of contribution to the PC1 was set and metabolites that satisfied this criterion were highlighted in red (Figure S1 and Table S1). Analysis was performed using RStudio software, Inc. Version 1.0.153 https://rstudio.com. All the data analyzed in this study fulfilled the criteria for normal distribution testing, using a D’Agostino and Pearson omnibus normality test and assessed for the homogeneity of the variance, thus parametric tests were used for all statistical comparisons. Statistical analyses were performed with GraphPad Prism 5.01 (GraphPad Software Inc. https://www.graphpad.com). Differences between groups were determined by Student’s t test (to compare 2 groups) or ANOVA (with Fisher protected least square as the post-hoc test, to compare n > 2 groups) and *p*-values < 0.05 were considered statistically significant.

## 5. Conclusions

In conclusion, the present findings suggest that TLR4 represents a potential mechanism of PI resistance by controlling mitochondrial fitness and its inhibition could be successful in patients with refractory MM to overcome bortezomib resistance.

## Figures and Tables

**Table 1:** Clinical characteristics of patients (pts) included in the study. Ref: refractory to BTZ and lenalidomide treatments.

pts	Sex	Age	Status Disease	Ig Type	R-ISS Stage	LDH (U/L)	Hb (g/dL)	ANC/mmc	ALC/mmc	AMC/mmc	k-lambda sFLC Ratio	FISH
*p#1*	M	49	Ref	IgAlambda	1	213	11.6	900	540	800	0.01	t (4;14)
*p#2*	M	72	Ref	IgGk	3	315	9.8	1670	560	870	230	t (11;14)
*p#3*	M	58	Ref	IgGk	3	280	9.7	1890	700	910	120	t (4;14)
*p#4*	M	73	Ref	IgGk	3	654	6.5	310	560	650	210	del17p13
*p#5*	F	68	Ref	IgGlambda	3	310	8.9	1100	780	760	0.01	del17p13

LDH: lactate dehydrogenase; Hb: hemoglobin; ANL: absolute neutrophil count; ALC: absolute lymphocyte count; AMC: absolute monocytic count; sFLC: serum Free Light Chains.

## Supplementary Materials

The following are available online at https://www.mdpi.com/2072-6694/12/8/1999/s1, Figure S1: Basal levels of TLR4 expression in HMCL, Figure S2: Principal Component Analysis (PCA) on metabolites levels highlights major contributors to discriminate among groups, Figure S3: TAK-242/BTZ combination showed a favorable cytoxicity profile toward CD138- cells, Table S1: Metabolites contribution to PCs expressed as squared cosine.
