# Supplementary material for: Inhibition of TLR4 Signaling Affects Mitochondrial Fitness and Overcomes Bortezomib Resistance in Myeloma Plasma Cells
Source: Cancers (Basel). 2020 Jul 22;12(8):1999. doi: 10.3390/cancers12081999 (PMC7463509; doi:10.3390/cancers12081999)
Supplement: Supplementary file 1 [file cancers-12-01999-s001.zip › supporting information/cancers-830300-supplementary-resubmit.docx]

Supplementary Materials

**
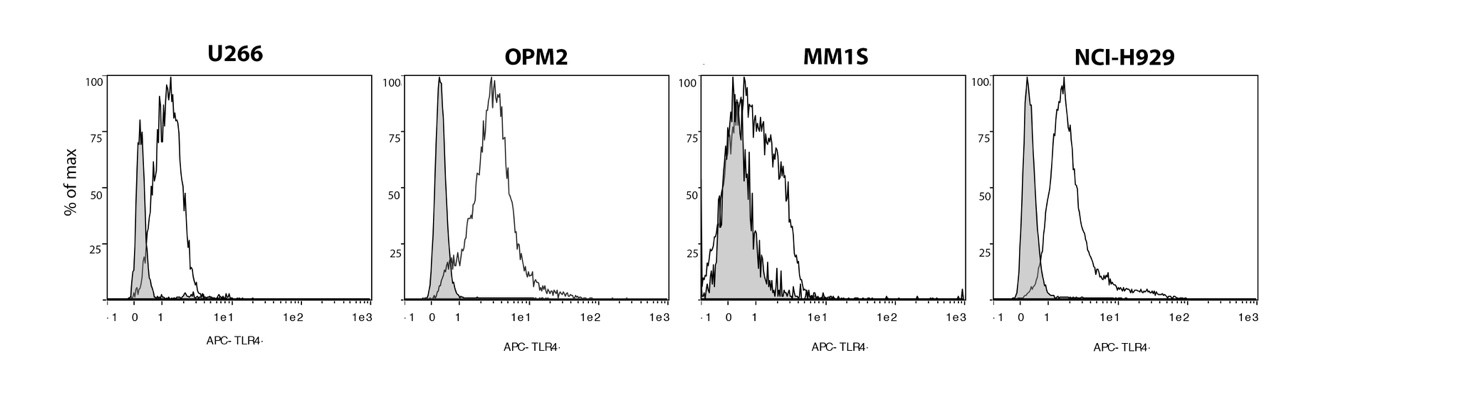
Supplementary Figure 1:** Basal levels of TLR4 expression in HMCL. Representative mean fluorescence intensity (MFI) values for APC conjugated anti-human TLR4 antibody binding to various MM cell lines (black line); gray area: isotype control.


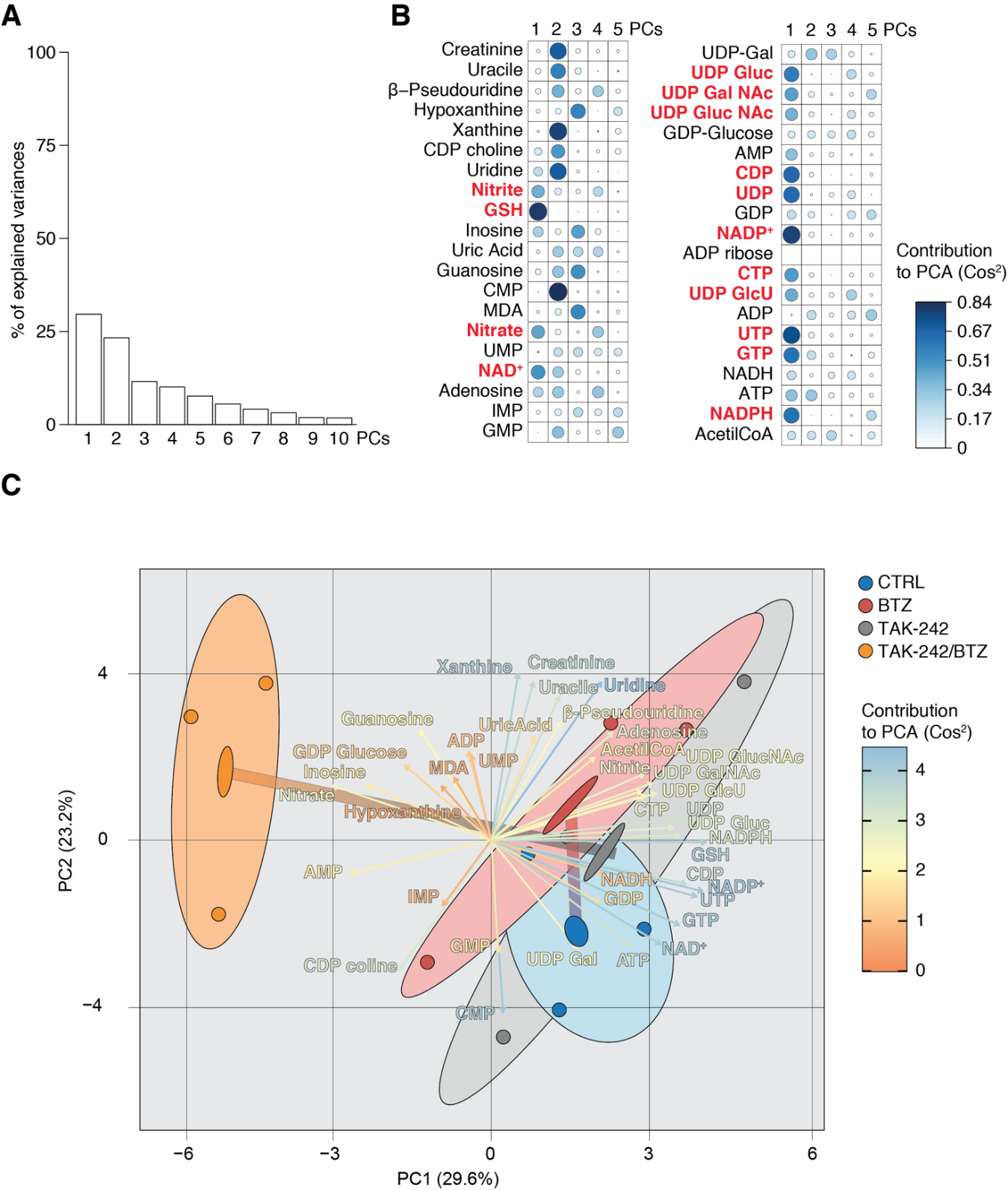


**Supplementary Figure 2:** Principal Component Analysis (PCA) on metabolites levels highlights major contributors to discriminate among groups. **A**) Scree plot of PCs (PC1-PC10) of the percentage of explained variance among metabolites. **B**) Quality of representation of variables on the factor map expressed as squared cosine (Cos2) of contribution to PCA; in red metabolites with squared cosine of contribution > 0.50 (see also Table 1). **C**) PCA biplot of metabolites levels in CTRL, BTZ, TAK-242 and TAK-242/BTZ group; key coloured arrows indicate the contribution of variables to the PCs; small ellipses represent the mean point of each group and confidence ellipses are also shown.

**
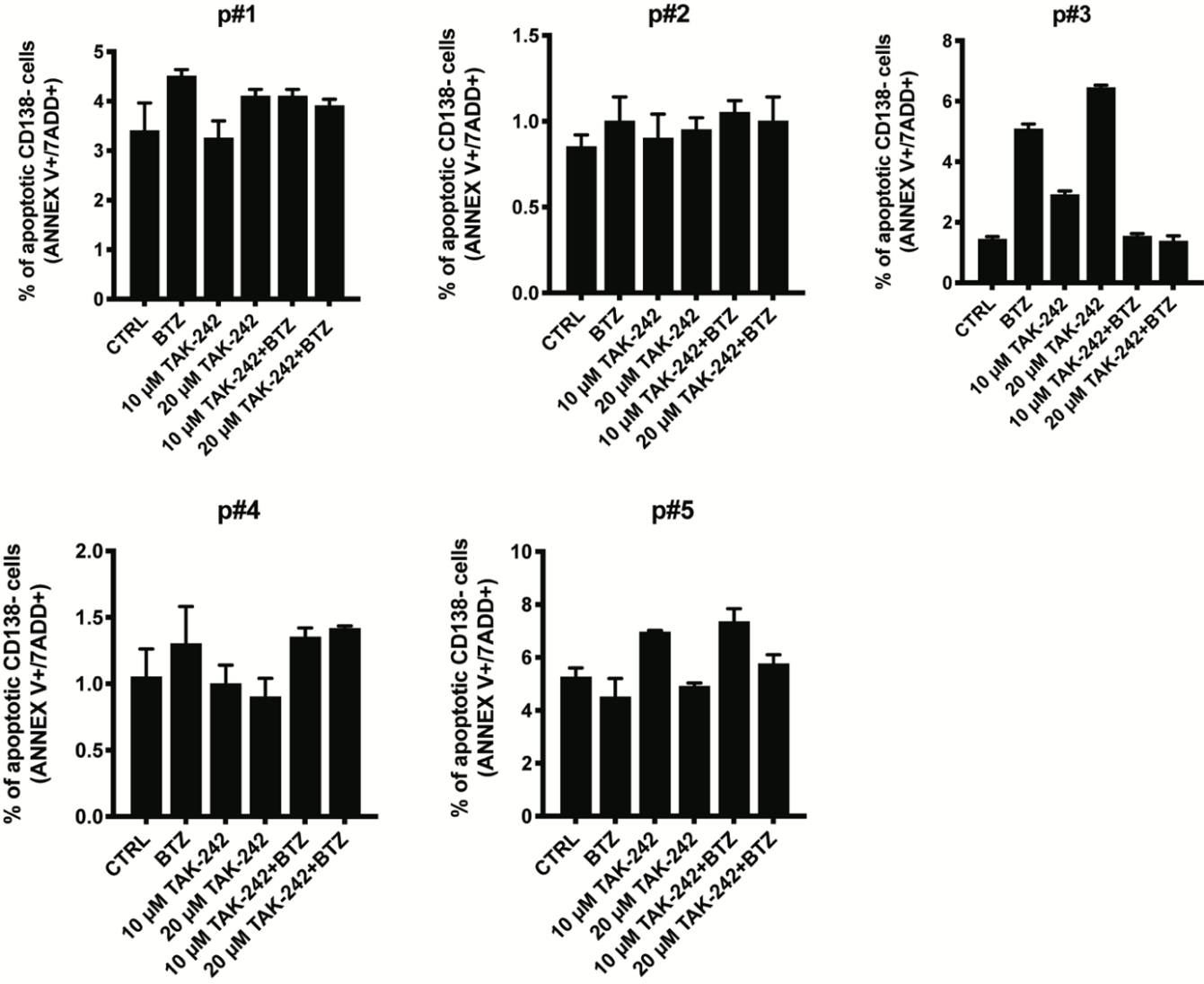
Supplementary Figure 3:** TAK-242/BTZ combination showed a favorable cytoxicity profile toward CD138- cells. Cell viability was measured by Annexin V/7-ADD staining-flow cytometry 48h post-treatment. Bars indicate the standard error means. **p* < 0.05; ***p* <0.01; ****p* < 0.001.

**Supplementary Table 1.** Metabolites contribution to PCs expressed as squared cosine.

| **Metabolites** | **PC1** | **PC2** | **PC3** | **PC4** | **PC5** |
| --- | --- | --- | --- | --- | --- |
| Creatine | 0.082 | 0.656 | 0.055 | 0.018 | 0.025 |
| Uracile | 0.193 | 0.476 | 0.115 | 0.005 | 0.000 |
| β-pseudouridine | 0.132 | 0.325 | 0.219 | 0.179 | 0.063 |
| Hypoxanthine | 0.032 | 0.120 | 0.501 | 0.064 | 0.224 |
| Xanthine | 0.069 | 0.709 | 0.003 | 0.006 | 0.074 |
| CDP-choline | 0.240 | 0.405 | 0.001 | 0.018 | 0.069 |
| Uridine | 0.340 | 0.545 | 0.028 | 0.009 | 0.005 |
| **Nitrite** | 0.506 | 0.044 | 0.006 | 0.234 | 0.008 |
| **GSH** | 0.811 | 0.023 | 0.001 | 0.001 | 0.002 |
| Inosine | 0.207 | 0.160 | 0.537 | 0.014 | 0.000 |
| Uric Acid | 0.081 | 0.270 | 0.371 | 0.118 | 0.020 |
| Guanosine | 0.036 | 0.425 | 0.489 | 0.013 | 0.002 |
| CMP | 0.008 | 0.853 | 0.001 | 0.010 | 0.006 |
| MDA | 0.008 | 0.152 | 0.517 | 0.006 | 0.026 |
| Nitrate | 0.347 | 0.166 | 0.000 | 0.350 | 0.005 |
| UMP | 0.000 | 0.239 | 0.103 | 0.244 | 0.181 |
| NAD^+^ | 0.405 | 0.429 | 0.099 | 0.000 | 0.031 |
| Adenosine | 0.360 | 0.213 | 0.000 | 0.369 | 0.004 |
| IMP | 0.062 | 0.086 | 0.350 | 0.046 | 0.164 |
| GMP | 0.009 | 0.367 | 0.010 | 0.058 | 0.278 |
| **UDP-Gal** | 0.562 | 0.022 | 0.058 | 0.003 | 0.075 |
| **UDP-Gluc** | 0.595 | 0.003 | 0.028 | 0.023 | 0.033 |
| **UDP-GalNac** | 0.509 | 0.196 | 0.008 | 0.019 | 0.138 |
| **UDP-GlucNac** | 0.704 | 0.105 | 0.013 | 0.000 | 0.001 |
| GDP-Glucose | 0.090 | 0.194 | 0.258 | 0.111 | 0.100 |
| AMP | 0.358 | 0.010 | 0.032 | 0.004 | 0.004 |
| **CDP** | 0.611 | 0.141 | 0.000 | 0.019 | 0.006 |
| **UDP** | 0.668 | 0.000 | 0.013 | 0.231 | 0.015 |
| GDP | 0.140 | 0.165 | 0.023 | 0.206 | 0.325 |
| **NADP^+^** | 0.661 | 0.176 | 0.010 | 0.013 | 0.012 |
| ADP-ribose | 0.000 | 0.000 | 0.000 | 0.000 | 0.000 |
| CTP | 0.456 | 0.015 | 0.002 | 0.023 | 0.001 |
| UDP-GlcU | 0.437 | 0.021 | 0.006 | 0.386 | 0.059 |
| ADP | 0.000 | 0.230 | 0.120 | 0.109 | 0.315 |
| **UTP** | 0.632 | 0.180 | 0.014 | 0.033 | 0.009 |
| GTP | 0.497 | 0.315 | 0.037 | 0.022 | 0.089 |
| NADH | 0.228 | 0.055 | 0.065 | 0.289 | 0.043 |
| ATP | 0.228 | 0.409 | 0.047 | 0.108 | 0.036 |
| **NADPH** | 0.556 | 0.010 | 0.001 | 0.026 | 0.257 |
| AcetilCoA | 0.291 | 0.108 | 0.200 | 0.042 | 0.153 |
