# Supplementary figures and images for: Inhibition of TLR4 Signaling Affects Mitochondrial Fitness and Overcomes Bortezomib Resistance in Myeloma Plasma Cells
Source: Cancers (Basel). 2020 Jul 22;12(8):1999. doi: 10.3390/cancers12081999 (PMC7463509; doi:10.3390/cancers12081999)

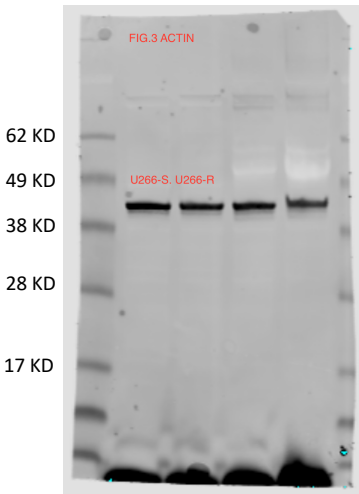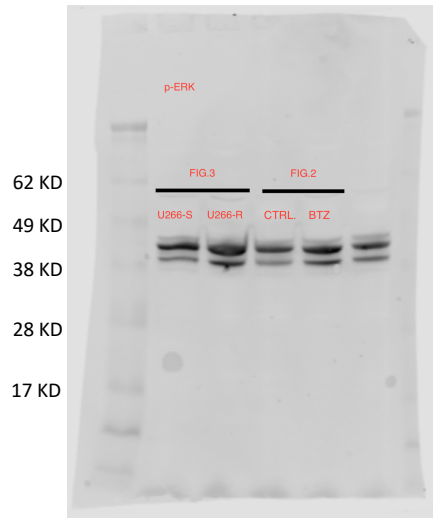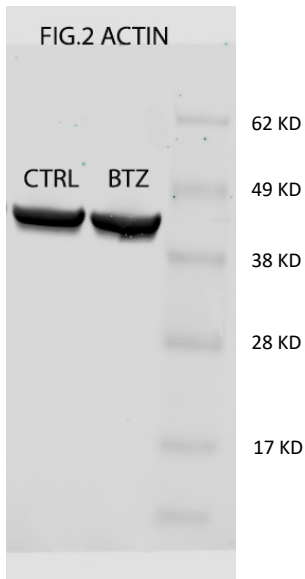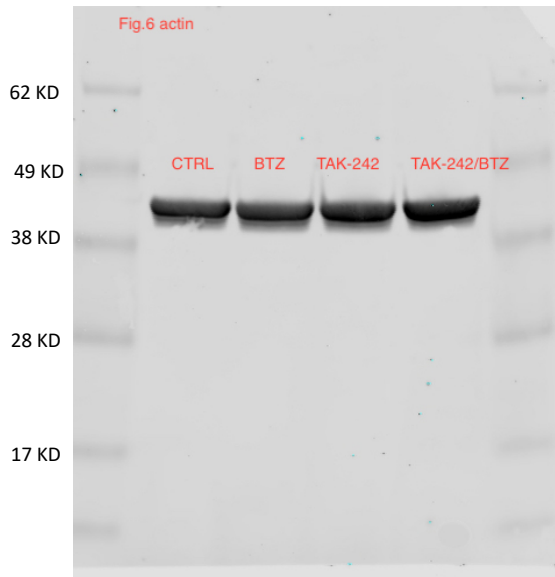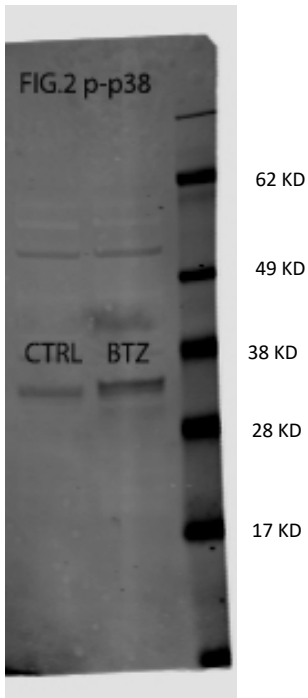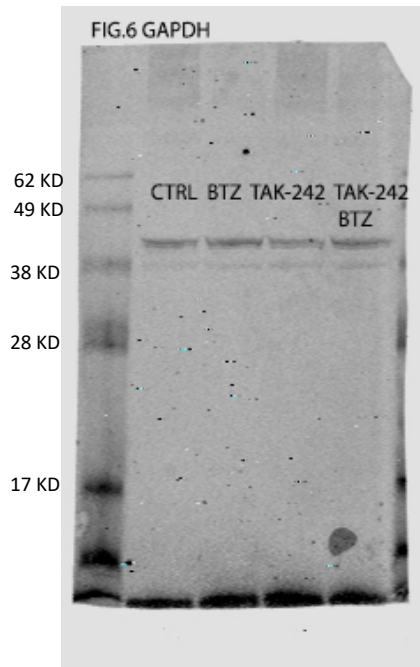

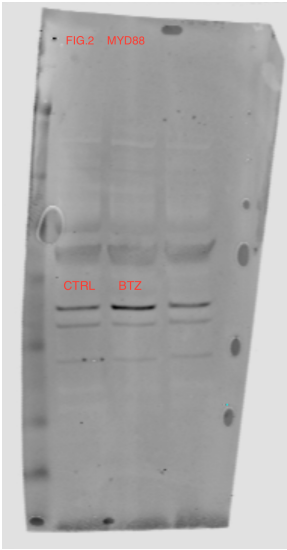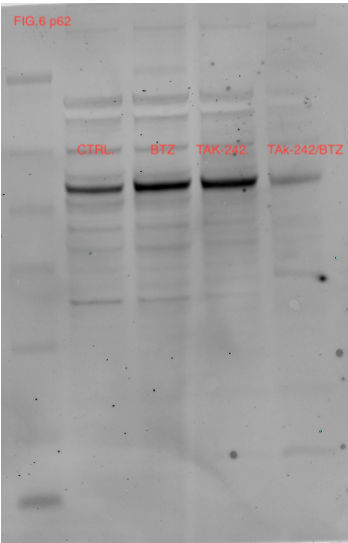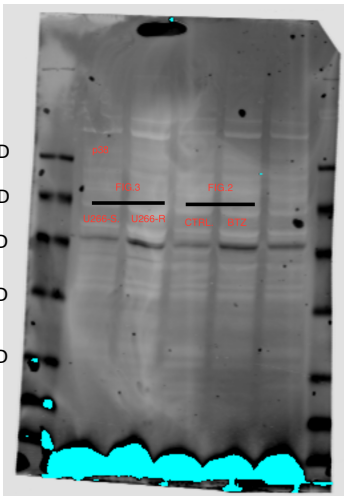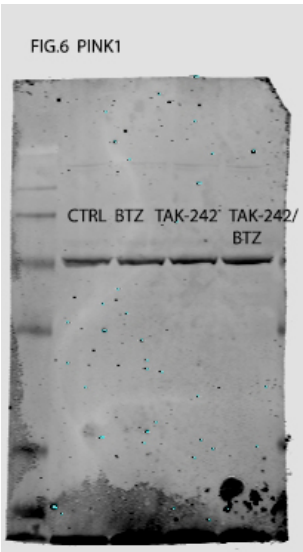

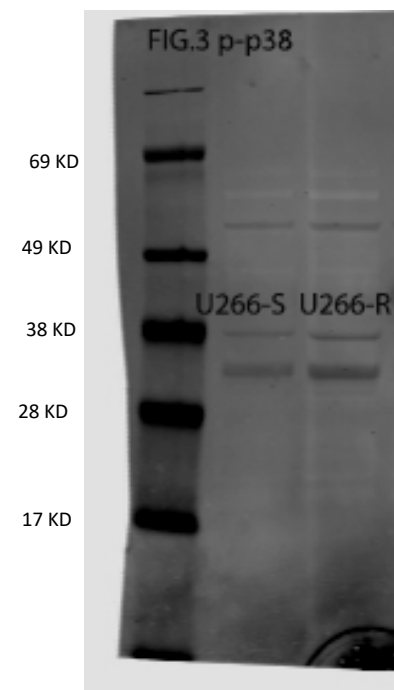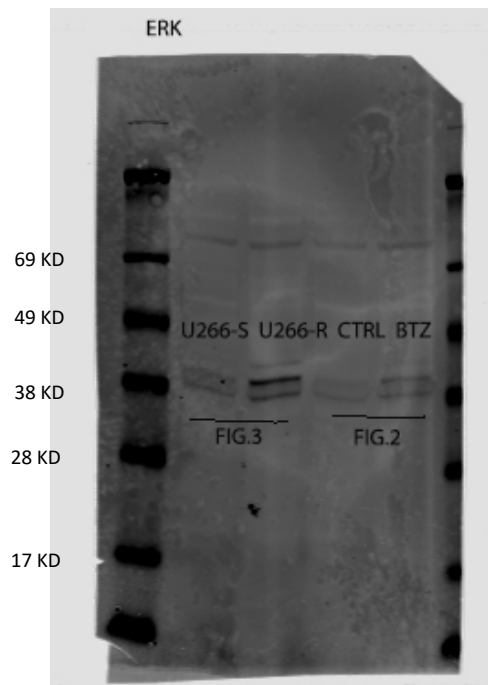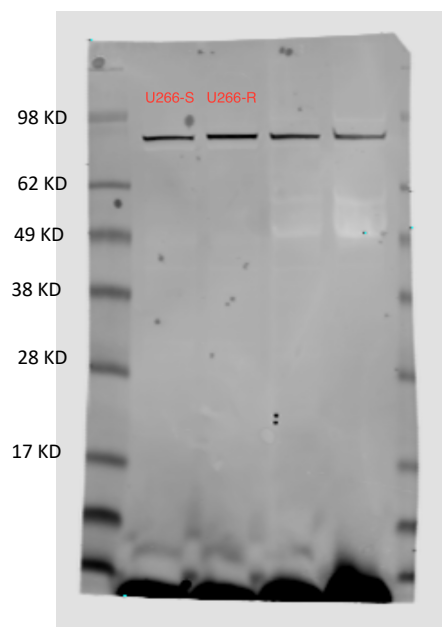

Supplement: Supplementary file 1 [file cancers-12-01999-s001.zip › supporting information/supplementary material.pdf]
